# Exploring the Interplay Between Senescent Osteocytes and Bone Remodeling in Young Rodents

**DOI:** 10.1155/2024/4213141

**Published:** 2024-11-16

**Authors:** Insun Song, Pil-Jong Kim, Yong Jun Choi, Yoon-Sok Chung, Soonchul Lee, Jeong-Hwa Baek, Kyung Mi Woo

**Affiliations:** ^1^Dental Research Institute, Seoul National University, Seoul 08826, Republic of Korea; ^2^School of Dentistry and Dental Research Institute, Seoul National University, Seoul 08826, Republic of Korea; ^3^Department of Endocrinology and Metabolism, Ajou University School of Medicine, Suwon 16499, Republic of Korea; ^4^Department of Orthopaedic Surgery, CHA Bundang Medical Center, CHA University School of Medicine, 59 Yatap-ro, Bundang-gu, Seongnam-si 13496, Republic of Korea; ^5^Department of Molecular Genetics, School of Dentistry and Dental Research Institute, Seoul National University, Seoul 08826, Republic of Korea

## Abstract

This study identifies senescent osteocytes in the femur and tibia of young rodents and explores their role in bone remodeling. The proximity of osteoclasts to senescent osteocytes was observed, which is a new finding. Cultured osteocytes, sorted using a podoplanin antibody in FACS, exhibited osteocytic characteristics and increased senescence-related genes. Senescent osteocytes secreted cytokines associated with senescence, remodeling, and inflammation. Notably, IGF1 and MMP2 were elevated in podoplanin-positive (pdpn^+^) osteocytes. Migration assays demonstrated significant osteoclast precursor migration towards senescent osteocytes, further confirmed by co-culture experiments leading to osteoclast differentiation. These findings suggest that senescent osteocytes have a pivotal role in initiating bone resorption, with recruitment of osteoclast precursors during early bone remodeling stages. In conclusion, our research enhances our understanding of complicated bone remodeling mechanisms and bone homeostasis.

## 1. Introduction

Bone remodeling is a dynamic and essential process for maintaining mineral homeostasis, repairing damaged bone, and ensuring the integrity, strength, and density of the skeleton [1–3]. This intricate process involves several sequential phases, including activation/initiation of resorption, resorption, transition/reversal, and formation/termination [1–3]. Bone remodeling relies on the interplay between three key cellular players: osteocytes, osteoclasts, and osteoblasts. These cells regulate bone remodeling and contribute significantly to maintaining plasma calcium homeostasis in the body [4].

During the activation phase of bone remodeling, various initiating signals, such as parathyroid hormone (PTH), estrogen, Bax, and Bcl-2, are detected in response to systemic changes in homeostasis [1, 5–9]. However, the precise mechanism underlying this phase remains elusive. This study investigated a new aspect of bone remodeling by examining the involvement of endogenous senescent osteocytes during the activation phase, specifically focusing on the early resorption phase.

Osteocytes, the most abundant cells in bones, have a crucial role in communicating with osteoclasts and osteoblasts through distinct signaling molecules [10–12]. The secretion of a higher amount of RANKL by osteocytes is of particular importance, which is known to be pivotal in bone remodeling [13]. Notably, osteocyte-derived RANKL has been linked to age-related cortical bone loss and induced by cellular senescence [14].

To increase our understanding of osteocyte-associated bone remodeling, we proposed a novel hypothesis on the recruitment of osteoclast precursors, or monocytes, to old bone sites for remodeling in young rodents. These osteoclast precursors originate from bone marrow progenitors and travel through the bloodstream to reach their target tissues. The recruitment process involves various factors, including chemotaxis, cytokines and specific molecules like type 1 collagen, osteocalcin, stromal cell-derived factor-1 (SDF-1), and monocyte chemoattractant protein-1 (MCP-1) [15–17].

Moreover, previous studies by Hayflick and Moorhead have demonstrated that limited cell division in primary cells leads to replicative cellular senescence [18]. This senescence is triggered by various stressors such as telomere shortening, oxidative stress, DNA damage, and oncogene activation [19–21]. Senescence cells release a specific secreted phenotype known as the senescence-associated secreted phenotype (SASP) that acts as a signal to the immune system, stimulating tissue repair in the damaged area and initiating the clearance of senescent cells [22–31]. The elimination of senescent cells plays a vital role in tissue remodeling and damage resolution [32–35]. Despite the complexity of cellular senescence, this study provides potential insights into defining the senescence status of osteocytes in young rodents. It identifies the triggers for the recruitment of osteoclast precursors. The findings of this study are highly relevant to the early stage of bone remodeling, for which we hypothesize that only senescent osteocytes actively participate, ultimately contributing to the sequential bone remodeling process. Therefore, our study proposes a new perspective on bone remodeling, focusing on the involvement of senescent osteocytes in the recruitment of osteoclast precursors during the early resorption phase.

## 2. Materials and Methods

### 2.1. Animals and Guidelines

Animals used in this study included 12-week-old female Sprague Dawley (SD) rats and 8∼10-week-old male C57BL/6 mice obtained from Orient Bio Inc (South Korea). They were housed in ventilated cages at a constant temperature and provided water and food *ad libitum*. The Institutional Animal Care and Use Committee (IACUC) approved all animal protocols (GEN-IACUC-1910-01), and all experiments followed the IACUC guidelines to ensure ethical treatment and adherence to internationally recognized principles of animal research.

### 2.2. Senescence-Associated Beta-Galactosidase (SA*β*G) and Tartrate-Resistant Acid Phosphatase (TRAP) Staining

For the detection of SA*β*G in the femur and tibia of SD rats, frozen samples were prepared after euthanasia. The tissues were fixed in a solution containing 0.2% glutaraldehyde, 2 mM MgCl_2_, 5 mM ethylenediaminetetraacetic acid (EDTA), and 0.02% NP40 in PBS at 4°C for 24 h. Subsequently, decalcification was performed in a 19% EDTA solution at 4°C for 10 days, followed by equilibration in 30% sucrose in PBS at 4°C for 24 h. The samples were embedded in Optimal Cutting Temperature (OCT) medium and cut into sections using cryomicrotomes with a tungsten carbide blade. For SA*β*G staining of the frozen sections, they were incubated in a SA*β*G solution containing 1 mg/mL X-gal (5-bromo-4-chloro-3-indolyl-D-b-galactoside), 5 mM potassium ferrocyanide, 5 mM potassium ferricyanide, 150 mM NaCl, and 2 mM MgCl_2_ at pH 6.0 for 1 day in a 37°C incubator. To detect SA*β*G in paraffin-embedded samples, the tissues were fixed in the same manner as the frozen samples, followed by SA*β*G staining with whole bone at 37°C for 1 day before decalcification in 19% EDTA at 4°C for 10 days. After these tissue processing steps, paraffin-embedded samples were sectioned, and SA*β*G staining was performed again with eosin counterstaining. TRAP activity, a marker for osteoclasts, was also stained using the TRAP staining kit (#AK04F, Cosmo Bio Co., LTD) to identify co-staining between X-gal (SA*β*G) and TRAP. Cell numbers and areas were measured using CaseViewer 2.4 and ImageJ software on scanned tissue slides.

### 2.3. Isolation of the Osteocytes From Mouse Femur and Tibia

**Figure 1:**
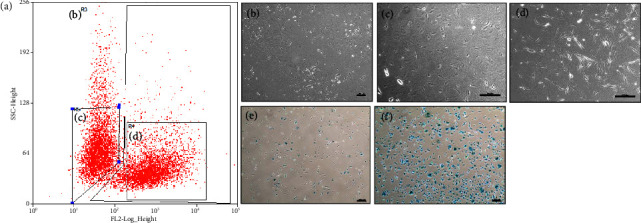
Fluorescence-activated cell sorting (FACS) of primary osteocytes by anti-podoplanin antibody and SA*β*G staining. (a) Sorted osteocytes are shown in regions R5 and R4, which represent a distinct cell population. (b) Total cells are shown in region R3 before sorting. (c) pdpn^−^ cells are represented in region R5. (d) pdpn^+^ cells are shown in region R4. (e) Depicts cells that are pdpn^−^ and less stained with SA*β*G. (f) Depicts cells that are both pdpn^+^ and SA*β*G^+^. Scale bar (b–f) = 200 *μ*m.

The mice were euthanized, and the femurs and tibias were collected for osteocyte preparation. Soft tissues and connective tissues were carefully removed from the harvested bones. We followed a modified version of the “Osteocyte isolation and culture method” described by Shah et al. [36]. To remove the bone marrow, the top and bottom of the femurs and tibias were incised, and PBS buffer was used to flush the bone cavities. The bone was chopped into pieces less than 1 mm in size to extract osteocytes from the bone matrix, and 0.1% type 1 collagenase was added. The bone fragments were then incubated in a shaking incubator at 37°C for 2 h. The cells extracted from the bone were rinsed and filtered using a 40 *μ*m cell strainer. Subsequently, the isolated cells were cultured in a 37°C, 5% CO_2_ incubator. After the culture period, healthy osteocytes were successfully obtained (Figure 1(b)).

### 2.4. FACS Sorting of the Mouse Osteocytes

Extracted mouse osteocytes were cultured for 1 and 2 weeks to obtain osteocytes suitable for FACS sorting. The sorting was based on the expression of the cell surface marker podoplanin (pdpn). To this end, a fluorescently labeled antibody against podoplanin/E11 (# sc-53533 AF488, Santa Cruz Biotechnology) was utilized. The FACS sorting was performed using the BD FACSAria Fusion system (BD Biosciences), which enabled the separation of osteocytes into pdpn^+^ and pdpn^−^ populations. The sorted osteocytes were cultured for an additional 1 week to facilitate their recovery and stabilization after the FACS process. Following the post-sorting culture period, the gene expression profiles and cytokine levels of the sorted osteocytes were analyzed.

### 2.5. qPCR Analysis of the Osteocytes

Primary mouse osteocytes were cultured, and total mRNAs were extracted from the cells using the Trizol reagent (FAVORGEN, #FATRR001). The concentration and quality of the extracted mRNAs were determined using a NanoDrop 2.0 spectrophotometer (Thermo Scientific, Foster City, CA, USA). To synthesize cDNA, the extracted mRNAs were subjected to reverse transcription using the Enzo cDNA synthesis kit (#ENZ-KIT106-0200). The qPCR analysis was performed to assess the gene expression levels of specific targets. The qPCR reactions were done with the CFX Connect™ Real-Time PCR System from Bio-Rad Laboratories, Inc.  Table 1 presents the primer sequences used for the qPCR.

### 2.6. Mouse Cytokine Array of the Osteocytes

Following the manufacturer's instructions, we utilized the Mouse Cytokine array kit (96 targets, #ab193659, Abcam) to investigate the differential cytokine expression between the pdpn^+^ and pdpn^−^ osteocytes. Briefly, we blocked the cytokine array membranes (C3 and C4) using the provided blocking buffer for 30 min to prevent nonspecific binding. Subsequently, we incubated the membranes with the cultured media of the pdpn^+^ and pdpn^−^ osteocytes, which were collected from 1-week and 2-week culture supernatants, respectively. Following the incubation with the cell culture media, the membranes were washed to remove any unbound substances. Next, we incubated the membranes with a cocktail of biotin-conjugated antibodies, each targeting various individual cytokines. After incubation with the biotin-conjugated antibodies, the membranes were further incubated with HRP-conjugated streptavidin for 2 h at room temperature. This step facilitated the specific binding of the biotin-conjugated antibodies to their corresponding targets on the membranes. The cytokine array membranes were then subjected to chemiluminescence detection using the Fusion FX6.0 chemiluminescence system (Vilber). This detection method enabled us to visualize and quantify the presence of cytokines on the membranes. The following targets were analyzed: Axl, Bfgf, BLC (CXCL13), CD30 Ligand (TNFSF8), CD30 (TNFRSF8), CD40 (TNFRSF5), CRG-2, CTACK (CCL27), CXCL16, CD26 (DPPIV), Dtk, Eotaxin-1 (CCL11), Eotaxin-2 (MPIF-2/CCL24), E-Selectin, Fas Ligand (TNFSF6), Fc gamma RIIB (CD32b), Flt-3 Ligand, Fractalkine (CX3CL1), GCSF, GITR (TNFRSF18), GM-CSF, HGFR, ICAM-1 (CD54), IFN-gamma, IGFBP-2, IGFBP-3, IGFBP-5, IGFBP-6, IGF-1, IGF-2, IL-1 beta (IL-1 F2), IL-10, IL-12 p40/p70, IL-12 p70, IL-13, IL-15, IL-17A, IL-17 RB, IL-1 alpha (IL-1 F1), IL-2, IL-3, IL-3 R beta, IL-4, IL-5, IL-6, IL-7, IL-9, I-TAC (CXCL11), KC (CXCL1), Leptin, Leptin R, LIX (CXCL5), L-Selectin (CD62L), Lungkine (CXCL15), Lymphotactin (XCL1), MCP-1 (CCL2), MCP-5, M-CSF, MDC (CCL22), MIG (CXCL9), MIP-1 alpha (CCL3), MIP-1 gamma (CCL9), MIP-2(CXCL2), MIP-3 beta (CCL19), MIP-3 alpha (CCL20), MMP-2, MMP-3, Osteopontin (OPN, SPP1), Osteoprotegerin (OPG, TNFRSF11B), Platelet Factor 4 (CXCL4), Pro-MMP-9, P-Selectin, RANTES (CCL5), Resistin, SCF, SDF-1 alpha (CXCL12 alpha), Sonic Hedgehog N-Terminal (Shh-N), TNFRI (TNFRSF1A), TNFRII (TNFRSF1B), TARC (CCL17), I-309 (TCA-3/CCL1), TECK (CCL25), TCK-1 (CXCL7), TIMP-1, TIMP-2, TNF alpha, Thrombopoietin (TPO), TRANCE (TNFSF11/RANKL), TROY (TNFRSF19), TSLP, VCAM-1 (CD106), VEGF-A, VEGFR1, VEGFR2, VEGFR3, and VEGF-D.

### 2.7. Migration Assay of the BMMs/Osteoclast Precursors

Cell migration assays were conducted using Transwell™ Permeable Supports with a pore size of 5 *μ*m (Corning, Acton, MA, USA). A total of 3 × 10^4^ osteocytes or MLO-Y4 cells were seeded in the lower chamber of a 24-well plate and allowed to attach for 6 h. Subsequently, 3 × 10^4^ BMMs or Raw264.7 cells were seeded in the upper chamber. BMMs were obtained from bone marrow cells following a previously described method [37, 38]. The bone marrow cells were cultured in *α*-minimal essential medium (*α*-MEM) supplemented with 10% fetal bovine serum (FBS) and 5 ng/mL macrophage colony-stimulating factor (M-CSF) (#315-02, PeproTech) for 16 h. Non-adherent cells were then harvested and cultured for an additional 3 days with 30 ng/mL M-CSF to differentiate them into osteoclast precursors. The floating cells were removed, and the adherent cells/BMMs were used as osteoclast precursors. MLO-Y4 cells were treated with 100 *μ*M hydrogen peroxide (H_2_O_2_) to induce cellular senescence. After seeding, the Transwell chambers were incubated at 37°C for 24, 48, and 72 h enabling cell migration. After the designated incubation times, the migrated cells on the lower surface of the Transwell membrane were stained with a 1% aqueous solution of crystal violet (#V5265, Sigma-Aldrich). The stained cells were then counted using the ImageJ software. All experiments were performed in at least three independent replicates to ensure reliable data.

### 2.8. Co-Culture of the Osteocytes and BMMs/Osteoclast Precursors

Co-culture experiments were conducted using osteocytes and BMMs at a ratio of 1:5, respectively. To generate osteoclasts, BMMs were seeded at a density of 5 × 10^3^ cells per well in a 96-well plate. Osteocytes were seeded at a density of 1 × 10^3^ cells per well in the same 96-well plate without any additional reagents. The co-culture plates were incubated at 37°C with 5% CO_2_ for 21 days.

### 2.9. Statistical Analysis

All experimental data were expressed as the mean ± standard deviation from three independent experiments. Statistical analyses were conducted using the GraphPad Prism 5 software (GraphPad Software, Inc.). The Student's *t*-test was used to compare the differences between two groups, and a significance level of *p* < 0.01 was considered to indicate statistically significant results.

## 3. Results

### 3.1. Intense SA*β*G Staining Observed Adjacent to the Growth Plate

We conducted exploratory SA*β*G staining on cryosections of 8-week C57BL6 femur and tibia, revealing pronounced staining in the metaphysis next to the growth plates (Figures S1(a)–S1(c)). The SA*β*G^+^ cells in this region were presumed to be osteocytes and osteoclasts (Figure S1(c)). To obtain clearer images, we prepared paraffin-embedded femur and tibia samples from a 12-week-old SD rat, which provided clear visualization of SA*β*G^+^ osteocytes and osteoclasts in the trabecular bone and cortical bone (Figures S1(d)–S1(f)).

### 3.2. SA*β*G^+/−^ Osteocytes and Osteoclasts in the Trabecular Bone

**Figure 2:**
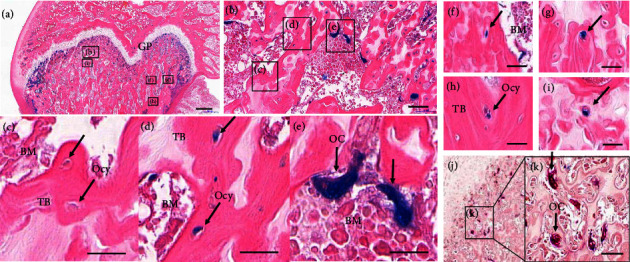
SA*β*G and TRAP staining of the trabecular bone in a 12-week SD rat femur. (a) SA*β*G and eosin staining of the epiphysis, growth plate and metaphysis. (b) Magnification of the region marked “(b)” in panel (a). (c) Magnification of the region marked “(c)” in panel (b) showing SA*β*G^−^ osteocytes (decline arrows). (d) Magnification of the region marked “(d)” in panel (b) showing SA*β*G^+^ osteocytes. (e) Magnification of the region marked “(e)” in panel (b) showing SA*β*G^+^ osteoclasts (vertical arrows). (f–i) SA*β*G and eosin staining of osteocytes in the trabecular bone distant from the growth plate. (j) SA*β*G and TRAP double staining (purple) in the metaphysis of the rat femur. (k) Magnification of the region “(k)” in panel (j) showing stained SA*β*G and TRAP (vertical arrows). Scale bar (a) = 500 *μ*m, (b) = 50 *μ*m, (c–k) = 20 *μ*m. (GP = growth plate, TRAP = tartrate-resistant acid phosphatase, Ocy = osteocyte, OC = osteoclast, TB = trabecular bone, BMMs = bone marrow-derived monocytes).

We observed SA*β*G^+/−^ osteocytes (Figures 2(a), 2(b), 2(c), 2(d), 2(e), 2(f), 2(g), 2(h), and 2(i)) in paraffin-embedded long bone tissues. SA*β*G^+^ osteocytes were found adjacent to the growth plate (Figure 2(a)) and at a distance from the growth plate (Figures 2(f), 2(g), 2(h), and 2(i)). Additionally, SA*β*G^+^ osteoclasts were detected at the periphery of the trabecular bone (Figures 2(e), 2(j), and 2(k)). SA*β*G^−^ osteocytes were also observed at some distance from the growth plate in the trabecular bone (Figure 2(c)). Notably, a significant population of intense SA*β*G^+^ osteocytes was detected near the growth plate (Figures 2(a), 2(b), and 2(d)) and as individual cells apart from other osteocytes (Figures 2(a), 2(f), 2(h), 2(h), and 2(i)). Double staining with SA*β*G and TRAP in the trabecular bone further supported these findings (Figures 2(j) and 2(k)). Previous studies by Colnot, Huang, and Helms, and Kopp et al. reported similar observations of SA*β*G^+^ osteoclasts [39, 40]. These data collectively indicate the presence of two distinct types of osteocytes in the trabecular bone: SA*β*G-positive (+) and SA*β*G-negative (−) osteocytes.

### 3.3. Investigating the Relationship Between SA*β*G^+^ Osteocytes and Osteoclasts in Proximity

**Figure 3:**
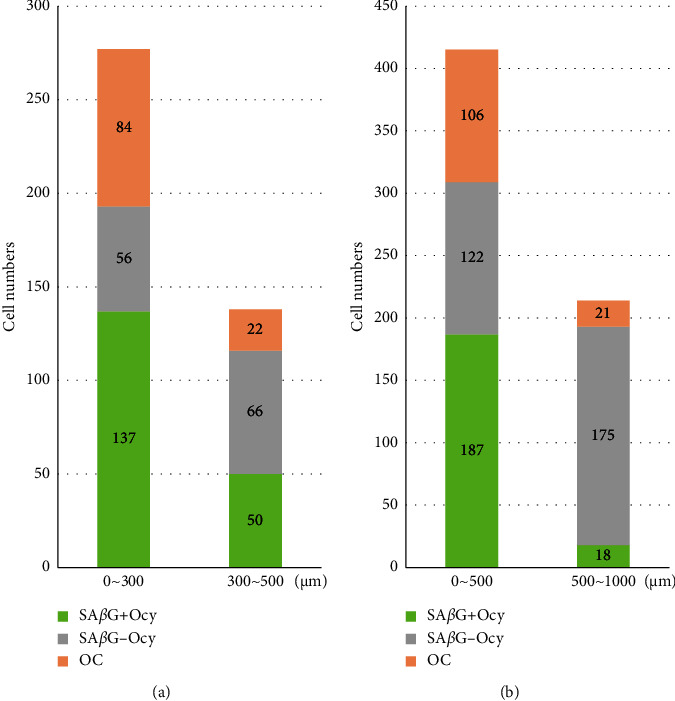
Cell number of SA*β*G^+/−^ osteocytes and osteoclasts within 1 mm from the growth plate. (a) Total number of SA*β*G^+/−^ osteocytes and osteoclasts within a distance range of 0∼300 *μ*m and 300∼500 *μ*m from the growth plate. (b) Total number of SA*β*G^+/−^ osteocytes and osteoclasts within a distance range of 0∼500 *μ*m and 500∼1000 *μ*m from the growth plate. Ocy = osteocytes, OC = osteoclasts.

To examine the expression patterns and association of SA*β*G in osteocytes and osteoclasts, we divided the areas around the growth plates into two different ranges: 0∼300 and 300∼500 *μ*m with a width of 450 *μ*m (Figure 3(a)). Additionally, we also divided into two different ranges: 0∼500 and 500∼1000 *μ*m with a width of 450 *μ*m (Figure 3(b)). We then quantified the number of SA*β*G^+/−^ osteocytes and SA*β*G^+^ osteoclasts in each area of each group. Within the range of 0–300 *μ*m, we observed 137 SA*β*G^+^ osteocytes, and 84 osteoclasts were detected compared to 56 SA*β*G^−^ osteocytes. However, in the 300–500 *μ*m range, the SA*β*G^+^ osteocytes and osteoclasts decreased to 50 and 22 cells, respectively, while SA*β*G^−^ osteocytes increased to 66 cells (Figure 3(a)). Similar patterns were observed in the 0 to 500 and 500–1000 *μ*m ranges. The total number of SA*β*G^+/−^ osteocytes and osteoclasts was 187/122 and 106 cells within 0–500 *μ*m, respectively. In contrast, 18/175 SA*β*G^+/−^ osteocytes and 21 osteoclasts were detected within the 500–1000 *μ*m range (Figure 3(b)). Overall, the SA*β*G^+^ osteocytes (187 ⟶ 18, 1/10) and osteoclasts (106 ⟶ 21, 1/5) were decreased within the 1000 *μ*m range, while more than 1.4 times of the SA*β*G^−^ osteocytes (122 ⟶ 175) were increased in the same region (Figure 3(b)). These data indicate that senescent phenotypic osteocytes are closely associated with osteoclasts compared to SA*β*G^−^ osteocytes.

### 3.4. pdpn^+^ Osteocytes Exhibit the SA*β*G^+^ Phenotype and a Higher Expression of Senescent Marker Genes

To study senescent osteocytes, we decided to culture the osteocytes for 1 and 2 weeks before FACS sorting. One-week cultured osteocytes showed no differences in SA*β*G staining between the pdpn^+^ and pdpn^−^ osteocytes (data not shown). Additionally, there were no significant differences in the expression of osteocyte marker genes and p16/CDKN2A between the pdpn^+^ and pdpn^−^ osteocytes (Figure S2).

**Figure 4:**
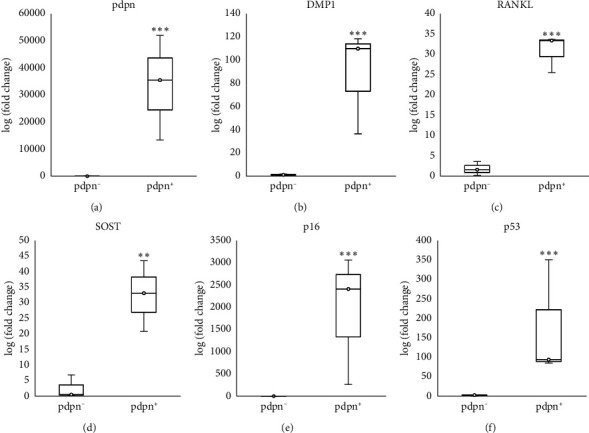
qPCR analysis of the osteocytes and senescence marker genes expression in pdpn^−^ and pdpn^+^ primary osteocytes cultured for 2 weeks before FACS sorting. (a) Expression of pdpn. (b) Expression of DMP1. (c) Expression of RANKL. (d) Expression of SOST. (e) Expression of p16/CDKN2A. (f) Expression of p53. Results are representative of at least three independent sets of similar experiments. *p* < 0.001^∗∗∗^, *p* < 0.01^∗∗^.

However, for the two-week cultured osteocytes, we observed a clear separation between the pdpn^+^ and pdpn^−^ osteocytes by FACS sorting (Figure 1). The majority of the pdpn^+^ cells expressed SA*β*G, and even a small proportion of SA*β*G^+^ cells were detected in the pdpn^−^ cells. Notably, osteocyte marker genes such as RANKL, DMP1, and SOST showed a higher expression in the pdpn^+^ osteocytes compared to the pdpn^−^ osteocytes in the two-week culture (Figures 4(b), 4(c), and 4(d)). On the other hand, other osteocyte marker genes such as keratocan, neuropeptide Y, matrix extracellular phosphoglycoprotein, phosphate regulating neutral endopeptidase on the chromosome X, and fibroblast growth factor 23 did not show any significant differences between the pdpn^+^ and pdpn^−^ osteocytes (data not shown). Of particular interest, the pdpn^+^ cells also exhibited a higher expression of senescence marker genes, including p16/CDKN2A and p53 (Figures 4(e) and 4(f)). These findings indicate that the pdpn^+^ osteocytes have robust osteocytic properties and senescent features.

### 3.5. Osteocyte-Related Cytokines in pdpn^+/−^ Osteocytes Such as SASP

**Figure 5:**
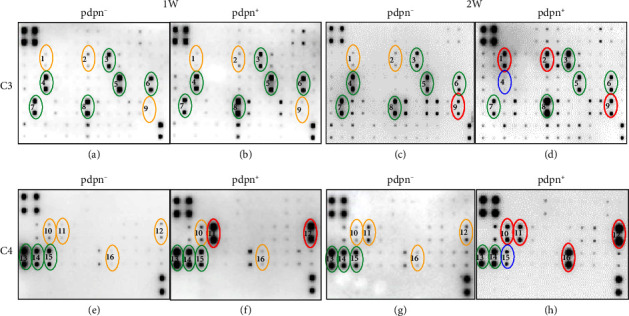
Cytokine array for SASP analysis of pdpn^+/−^ osteocyte supernatants. (a and b) Cytokines array of pdpn^−^ and pdpn^+^ osteocyte supernatants after a 1-week culture before FACS sorting (C3 membrane). (c and d) Cytokines array of pdpn^−^ and pdpn^+^ osteocyte supernatants after a 2-week culture before FACS sorting (C3 membrane). (e and f) Cytokines array of pdpn^−^ and pdpn^+^ osteocyte supernatants after a 1-week culture before FACS sorting (C4 membrane). (g and h) Cytokines array of pdpn^−^ and pdpn^+^ osteocyte supernatants after a 2-week culture before FACS sorting (C4 membrane). (1) CX3CL1, (2) IGFBP3, (3) IGFBP6, (4) IL6, (5) CXCL1/KC, (6) CXCL5/LIX, (7) CCL2/MCP1, (8) CCL9/MIP1*γ*, (9) RANTES/CCL5, (10) IGFBP2, (11) IGF1, (12) MMP2, (13) MMP3, (14) OPN/SPP1, (15) OPG/TNFRSF11B. (16) TIMP2. Red circles ⟶ increased expression compared to pdpn^−^ or 1-week membrane, Blue circles ⟶ decreased expression compared to pdpn^−^ or 1-week membrane, Green circles ⟶ almost the same expression between pdpn^+^ and pdpn^−^, and 1-week and 2-week membranes. Orange circles ⟶ lower expression than other membranes.

To identify SASP in pdpn^+/−^ osteocytes, we used a cytokine array with a mouse cytokine antibody array, investigating 96 mouse cytokines. More than 16 cytokines were detected through the cytokine array. In the groups cultured for 1 and 2 weeks, 9 and 10 cytokines were detected in the pdpn^−^ group, respectively, and 11 and 15 cytokines were observed in the pdpn^+^ group respectively (Table S1). Interestingly, IGF1 and MMP2 expression significantly increased in the pdpn^+^ group, while IGFBP6, IL6, CXCL1/KC, CXCL5/LIX, CCL2/MCP1, CCL9/MIP1r, MMP3, OPN/SPP1, and OPG/TNFRSF11B were expressed almost equally in both groups, pdpn^+^ and pdpn^−^ (Figures 5(a), 5(b), 5(e), and 5(f); Table S1). Interestingly, seven cytokines (CX3CL1, IGFBP3, RANTES/CCL5, IGFBP2, IGF1, MMP2, and TIMP2) were increased in the pdpn^+^ compared to the pdpn^−^ in the 2-week group. Among them, CX3CL1, IGFBP3, RANTES/CCL5, IGFBP2, and TIMP2 were newly expressed in pdpn^+^ in the 2-week group (Figures 5(c), 5(d), 5(g), and 5(h); Table S1). However, OPG and IL6 expression decreased or disappeared (Figures 5(f) and 5(h); Table S1). Taken together, these data suggest that the pdpn^+^ group secretes more senescence-dependent cytokines in remodeling and inflammation.

### 3.6. Pdpn^+^/SA*β*G^+^ Osteocytes Attract Osteoclast Precursors

**Figure 6:**
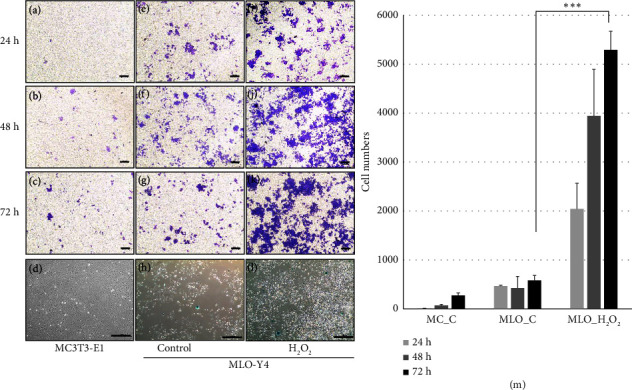
Migration assay of Raw264.7 in response to senescent MLO-Y4. (a–c), (e–g), and (i–k) Migration of Raw264.7 cells to MC3T3-E1 (negative control) and MLO-Y4 (control) at 24, 48, and 72 h, respectively. (d) SA*β*G staining of MC3T3-E1 (48-h cultured cells). (h) SA*β*G staining of MLO-Y4 (48-h cultured cells). (l) SA*β*G staining of MLO-Y4 treated with H_2_O_2_ (48-h cultured cells). (m) Migration cell number of Raw264.7. *p* < 0.001^∗∗∗^. Scale bar (a–c), (e–g), and (i–k) = 200 *μ*m. (d), (h), and (l) = 500 *μ*m. MC_C = MC3T3-E1. MLO_C = MLO-Y4. MLO_H_2_O_2_ = 100 *μ*M H_2_O_2_ treatment group.

**Figure 7:**
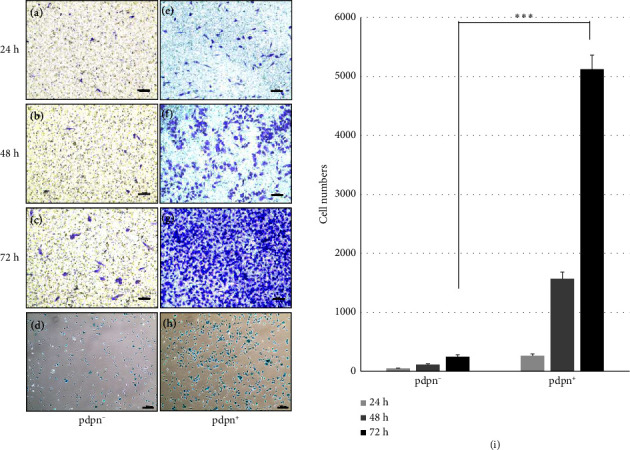
Migration assay of the bone marrow-derived monocytes (BMMs) in response to pdpn^+/−^ cells. (a–c) and (e–g) Monocyte migration to pdpn^+/−^ cells for 24, 48, and 72 h, respectively. (d) and (h) SA*β*G stained pdpn^+/−^ cells, indicating senescence. (i) Migration cell number of BMM. *p* < 0.001^∗∗∗^. Scale bar (a–c) and (e–g) = 200 *μ*m, (d) and (h) = 500 *μ*m.

We used a migration assay with Transwells to investigate the recruitment of osteoclast precursors to SA*β*G^+^ osteocytes. Initially, we treated the osteocyte cell line MLO-Y4 with 100 *μ*M H_2_O_2_ to induce senescence for 24, 48, and 72 h. After treatment, we assessed the migration of Raw264.7 cells toward the senescent osteocytes. (Figure 6(m)). Our observations revealed that senescence-induced osteocytes exhibited a higher attraction for Raw264.7 cells (Figures 6(i), 6(j), 6(k)) compared to the noninduced control (Figures 6(e), 6(f), 6(g)) and negative control (Figures 6(a), 6(b), 6(c)). The migration of Raw264.7 cells towards senescent osteocytes occurred in a time-course manner (Figure 6(m)). In addition, we examined the migration of primary osteoclast precursors towards SA*β*G^+/−^ osteocytes separated by antipodoplanin (Figure 7). Interestingly, the primary osteoclast precursors showed a greater tendency to migrate towards the pdpn^+^ osteocytes with a SA*β*G^+^ phenotype compared to the pdpn^−^ osteocytes. This difference was particularly significant at 72 h (Figure 7(g)). Similar results were observed in both the primary osteocytes and the cell line, indicating that the degree of osteocyte senescence influenced the migration of the osteoclast precursors (Figures 6(k) and 7(g)). Based on these findings, we proposed that senescent osteocytes can recruit osteoclast precursors, thus having a crucial role in bone resorption.

### 3.7. Co-Culture of pdpn^+^ Osteocytes Promotes Osteoclast Differentiation

**Figure 8:**
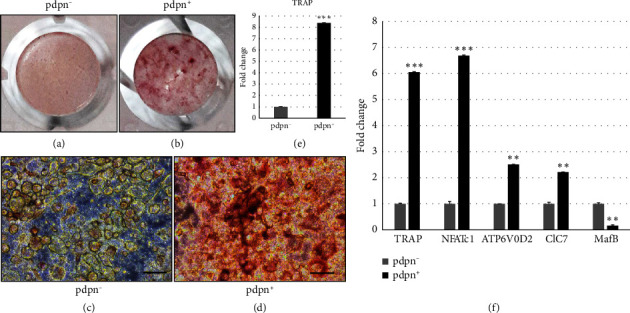
Co-culture of the osteocytes and BMMs for 3 weeks. (a) TRAP staining of the co-cultured pdpn^−^ osteocytes and BMMs. (b) TRAP staining of the co-cultured pdpn^+^ osteocytes and BMMs. (c) Microscope image showing TRAP staining of the pdpn-co-cultured cells. (d) Microscope image showing TRAP staining of the pdpn^+^ co-cultured cells. (e) Fold change of pdpn^+^ compared to pdpn^−^ TRAP^+^. (f) Osteoclast marker gene expression; tartrate-resistant acid phosphatase (TRAP), nuclear factor-activated T cells c1 (NFATc1), ATPase H^+^ transporting V0 subunit D2 (ATP6V0D2), chloride voltage-gate channel 7 (ClC7), MAF b ZIP transcription factor B (MafB). (a) and (b) Cultured in 96-well plates. Scale bar (c) and (d) = 50 *μ*m.

To investigate the potential of pdpn^+^ osteocytes in promoting osteoclast differentiation, we co-cultured BMMs with pdpn^+/−^ osteocytes for 21 days. The co-culture was designed to assess whether BMMs could differentiate into osteoclasts without the addition of M-CSF and RANKL. Encouragingly, we observed that the pdpn^+^ osteocytes more effectively induced the differentiation of osteoclast precursors into osteoclasts. This was evident as the pdpn^+^ osteocyte group exhibited over eight times more TRAP-stained cells compared to the pdpn^−^ group (Figures 8(b) and 8(d) and S3(b)). To further validate the osteoclast differentiation process in the pdpn^+/−^ groups, we analyzed the expression of osteoclast marker genes with qPCR. In the pdpn^+^ group, TRAP, NFATc1, ATP6V0D2 and ClC7 expression levels were significantly higher than in the pdpn^−^ group. Conversely, the negative marker MafB had a lower expression in the pdpn^+^ group compared to the pdpn^−^ group (Figure 8(f)). Taken together, these findings strongly suggest that pdpn^+^ osteocytes have a pivotal role in promoting osteoclast differentiation. Further investigation into the underlying mechanisms by which pdpn^+^ osteocytes facilitate this process could provide valuable insights into bone remodeling and tissue homeostasis regulation.

## 4. Discussion

In this study, we discover the presence of senescent osteocytes in the femur and tibia of young rodents (Figure 2) and explore their potential role in bone remodeling. We also observed the appearance of osteoclasts near senescent osteocytes. SA*β*G^+^ osteoclasts have been previously reported by Colnot, Huang, and Helms [39], and Kopp et al. [40]. Here, we detected SA*β*G^+^ osteoclasts and SA*β*G^+^ osteocytes in normal and young rodents, which was a novel finding. They were located near each other in the trabecular and cortical bone, especially next to growth plates (Figure 2(a)). To infer the relationship between osteocytes and osteoclasts, we quantified the number of SA*β*G^+/−^ osteocytes and osteoclasts within specific regions of the trabecular bone. Interestingly, the SA*β*G^+^ osteocytes had the same high or low number of cells as the SA*β*G^+^ osteoclasts (Figure 3), whereas the SA*β*G^−^ osteocytes appeared to have the opposite number of the SA*β*G^+^ osteocytes and osteoclasts. Consequently, we postulate that SA*β*G^+^ osteocytes are more closely associated with osteoclasts compared to SA*β*G^−^ osteocytes.

Next, we cultured the osteocytes for one and 2 weeks to obtain pure osteocytes and senescent osteocytes. Then, the cultured osteocytes were sorted with podoplanin antibody in a FACS machine and verified by qPCR. FACS-sorted pdpn^+^ cells exhibited osteocytic expression such as DMP1, RANKL, and SOST (Figures 4(b), 4(c), and 4(d)) and increased senescence-related genes including p16/CDKN2A and p53, compared with the pdpn^−^ cells (Figures 4(e) and 4(f)). These results indicate that 2-week cultured pdpn^+^ osteocytes show robust osteocytic characteristics along with senescent features.

Osteocytes secrete inflammatory cytokines that regulate the functions of other bone cells [41]. In this study, 16 classified cytokines are known to be involved in senescence, remodeling and inflammation (Table S1). Among them, 10 cytokines were reported in osteocytes (Table S1). IGF1 and MMP2 were commonly increased in the pdpn^+^ at 1- and 2-week samples. IGF1 is a well-defined molecule in bone remodeling, which is involved in the stimulation of osteoblasts and inhibition of osteoclasts, leading to enhanced bone growth, bone mass and bone repair [42–44]. MMP2 also has a pivotal role in bone and tissue remodeling involved in collagen degradation, osteoclast activation and activation of growth factors [45–48]. Among the 16 cytokines, CX3CL1, CXCL1, and CCL2 are associated with monocyte recruitment similar to SDF-1 [49–54]. Monocyte/macrophage recruitment is crucial in various biological processes, including wound healing and embryonic development, in which cellular senescence has also been implicated. We hypothesized that senescent osteocytes may have a similar role in bone remodeling. We performed a series of experiments to test our hypothesis, including migration assays and co-culture experiments with osteocytes and osteoclast precursors (Figures 6, 7, and 8). Our results consistently showed that osteoclast precursors significantly migrated toward senescent osteocytes in a time-course manner (Figures 6 and 7).

Additionally, co-culture experiments further substantiated our findings because we observed the differentiation of osteoclast precursors into osteoclasts in the presence of activated pdpn + osteocytes (Figures 8; S3). Based on these compelling results, we propose that senescent osteocytes are pivotal in initiating bone resorption during the activation phase of bone remodeling. Their ability to recruit osteoclast precursors and induce their differentiation into osteoclasts appears to be crucial for the precise localization of osteoclast precursors in old bone. This precise localization will likely facilitate the efficient replacement of old bone tissue with new bone.

We also analyzed the protein-protein interaction networks using the STRING database (https://string-db.org/) (Figures S4 and S5) to gain insights into the interactions and correlations between the detected cytokines. This analysis revealed that the SASP factors released by senescent osteocytes have the potential to attract monocytes and macrophages, which are known to be involved in various physiological processes, including wound healing and embryonic development in which cellular senescence also has crucial roles [22, 23, 55–59].

In conclusion, our research clarifies the role of senescent osteocytes in the early phases of bone remodeling. These senescent osteocytes have a significant role in promoting bone resorption by regulating the recruitment and differentiation of osteoclast precursors. The findings from this study have the potential to significantly impact our understanding of the fundamental mechanisms governing bone remodeling. Moreover, they offer promising avenues for future therapeutic interventions that address senescence-related bone disorders.

## Figures and Tables

**Table 1:** qPCR primer sequence.

Gene	Sequence
Pdpn-F	5′-AGC CGC TGT AGA ACC AAG AA-3′
Pdpn-R	5′-TTG GGT ACC AAC ACA GAC GA-3′
RANKL-F	5′-AGC CGA GAC TAC GGC AAG TA-3′
RANKL-R	5′-CCA CAA TGT GTT GCA GTT CC-3′
DMP1-F	5′-AGT CAG GCA GGA GAC CAA GAA AA-3′
DMP1-R	5′-TGG GTT TGT TGT GGT AAG CA-3′
SOST-F	5′-ACC CCG TGT AGA CTG GTG AG-3′
SOST-R	5′-ACA AGG ATG GGA GGT GAC TG-3′
p16-F	5′-ATC TGG AGC AGC ATG GAG TC-3′
p16-R	5′-TCG CAC GAT GTC TTG ATG TC-3′
p53-F	5′-CGG GTG GAA GGA AAT TTG TA-3′
p53-R	5′-CTT CTG TAC GGC GGT CTC TC-3′
TRAP-F	5′-CTG GAG TGC ACG ATG CCA GCG ACA-3′
TRAP-R	5′-TCC GTG CTC GGC GAT GGA CCA GA-3′
NFATc1-F	5′-CTC GAA AGA CAG CAC TGG AGC AT-3′
NFATc1-R	5′-CGG CTG CCT TCC GTC TCA TAG-3′
ATP6V0D2-F	5′-CAG ACG CGC TTT AAT CAT CA-3′
ATP6V0D2-R	5′-TTC GAT GCC TCT GTG AGA TG-3′
ClC7-F	5′-ATG ACC CAC CAG GCT CCT AT-3′
ClC7-R	5′-CCA CAG GGA ATC CGT TGT GA-3′
MafB-F	5′-CAT CAC CAT CAT CAC CAA GC-3′
MafB-R	5′-AGC TGC GTC TTC TCG TTC TC-3′
GAPDH-F	5′-TGA CCA CAG TCC ATG CCA TCA CTG-3′
GAPDH-R	5′-CAG GAG ACA ACC TGG TCC TCA GTG-3′

## Data Availability

The data that support the findings of this study are available from the corresponding author upon reasonable request.

## Supporting Information

Supporting Information Supporting Figure 1: SA*β*G staining of trabecular and cortical bone next to the growth plates in 8-week C57BL/6 and 12-week SD rats. Supporting Figure 2: qPCR analysis of the osteocytes and senescence marker genes expression of pdpn^−^ and pdpn^+^ in primary osteocytes cultured for 1 week before FACS sorting. Supporting Figure 3: TRAP staining of the osteoclasts in co-cultured osteocytes and BMMs for 3 weeks in 96-well plates. Supporting Figure 4: Multiple protein interactions of the pdpn^+/−^ cytokines after 1-week culture of osteocytes. Supporting Figure 5: Multiple protein interactions of the pdpn^+/−^ cytokines after 2-week culture of osteocytes. Table S1: Cytokines expression in 1- and 2-week pdpn ± membranes.
