# Supplementary material for: Exploring the Interplay Between Senescent Osteocytes and Bone Remodeling in Young Rodents
Source: J Aging Res. 2024 Nov 16;2024:4213141. doi: 10.1155/2024/4213141 (PMC11585373; doi:10.1155/2024/4213141)
Supplement: Supporting Information — Supporting Figure 1: SAβG staining of trabecular and cortical bone next to the growth plates in 8-week C57BL/6 and 12-week SD rats. Supporting Figure 2: qPCR analysis of the osteocytes and senescence marker genes expression of pdpn− and pdpn+ in primary osteocytes cultured for 1 week before FACS sorting. Supporting Figure 3: TRAP staining of the osteoclasts in co-cultured osteocytes and BMMs for 3 weeks in 96-well plates. Supporting Figure 4: Multiple protein interactions of the pdpn+/− cytokines after 1-week culture of osteocytes. Supporting Figure 5: Multiple protein interactions of the pdpn+/− cytokines after 2-week culture of osteocytes. Table S1: Cytokines expression in 1- and 2-week pdpn ± membranes. [file 4213141.f1.zip › 4213141 supplementary 2024-09-22.docx]

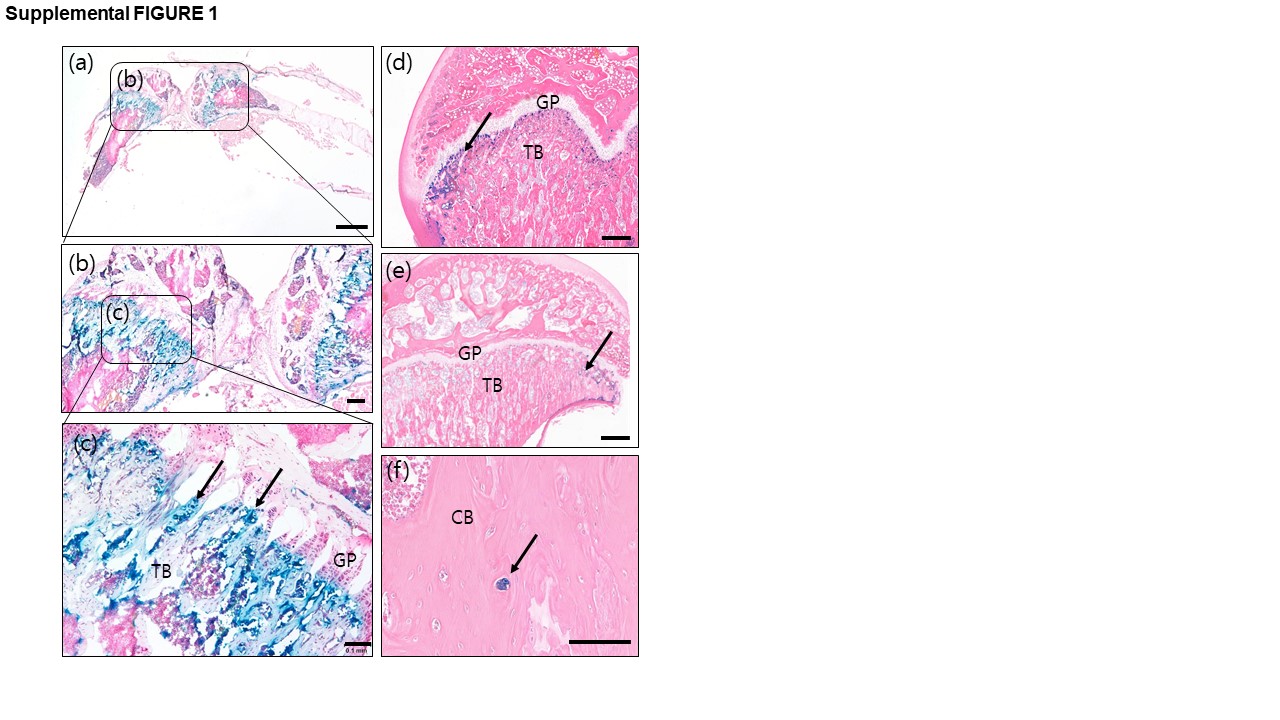


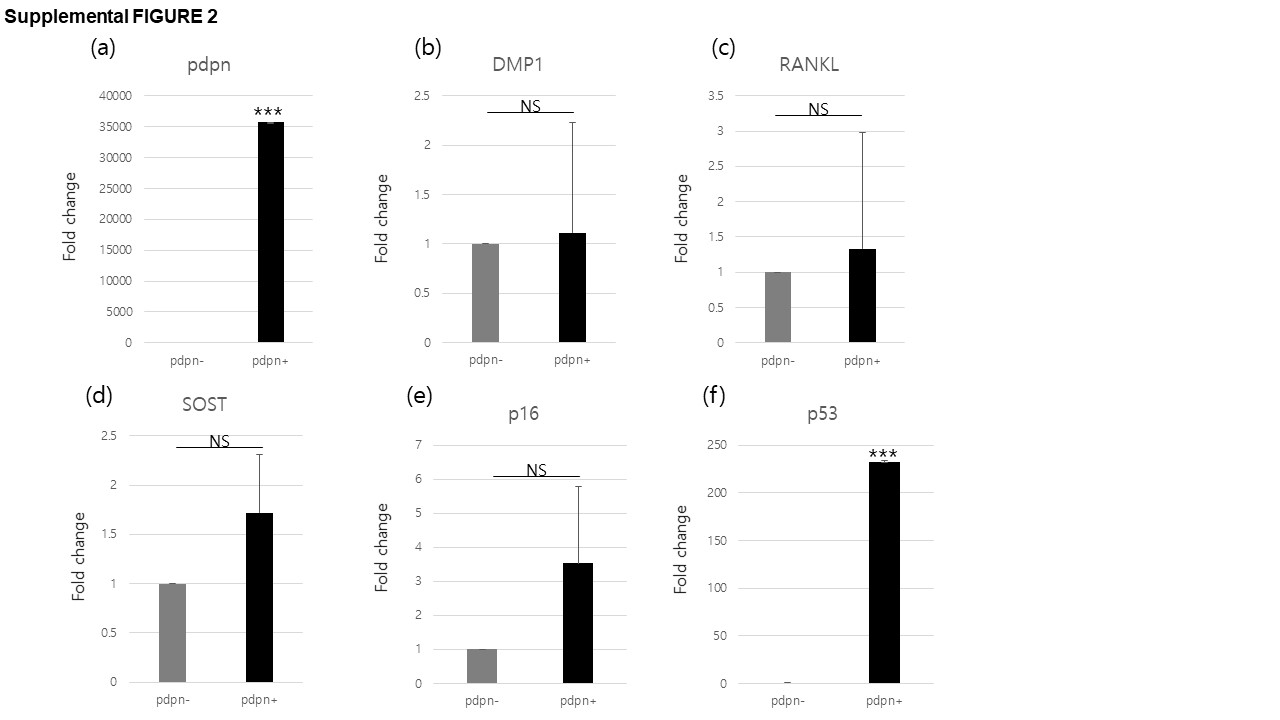


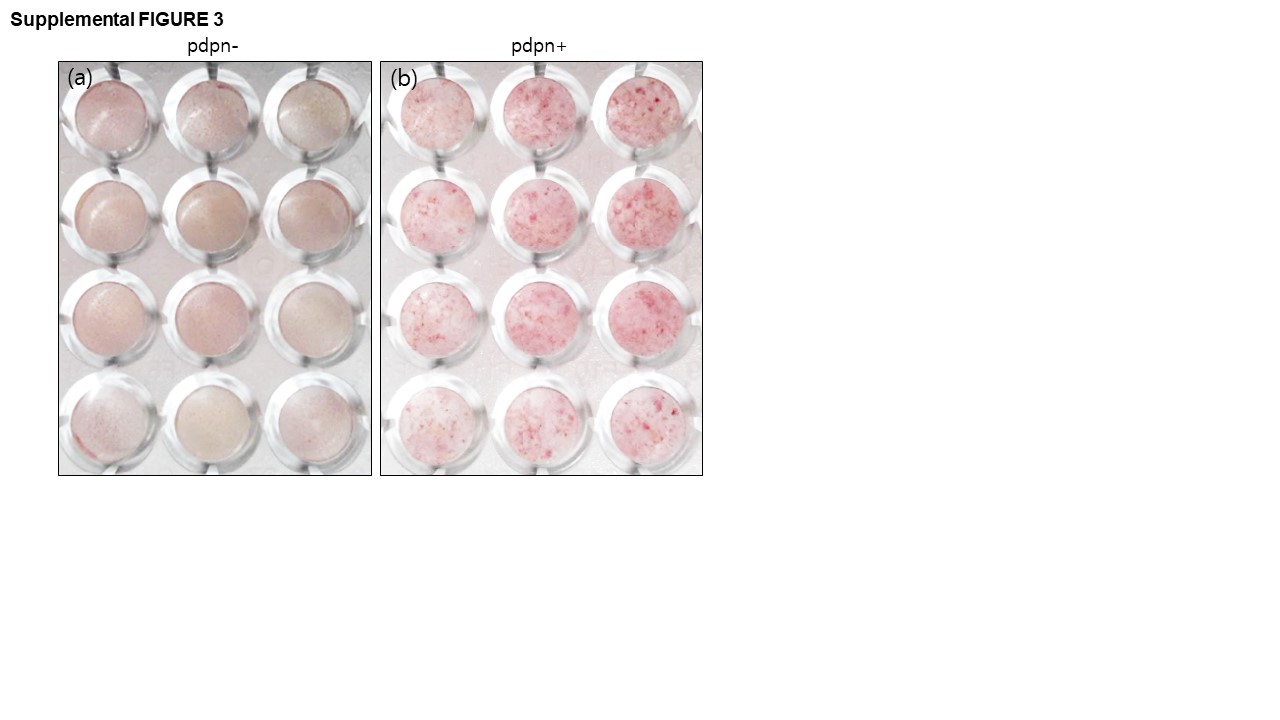


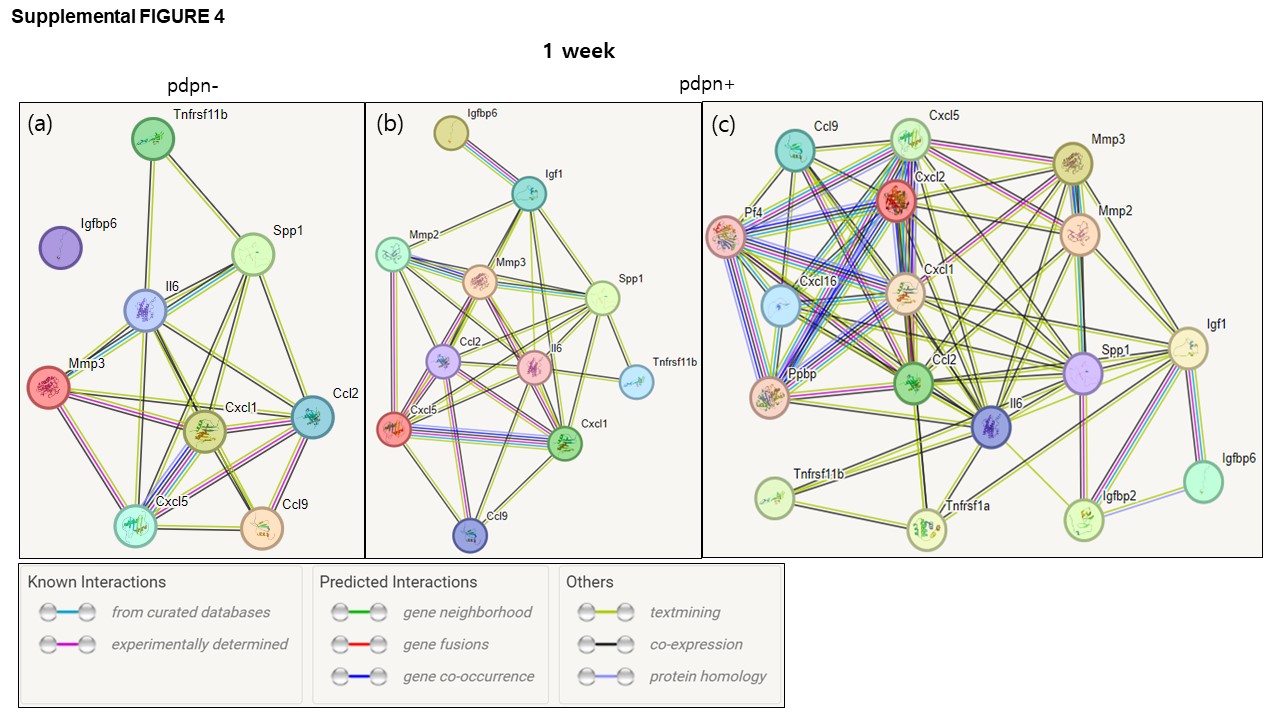


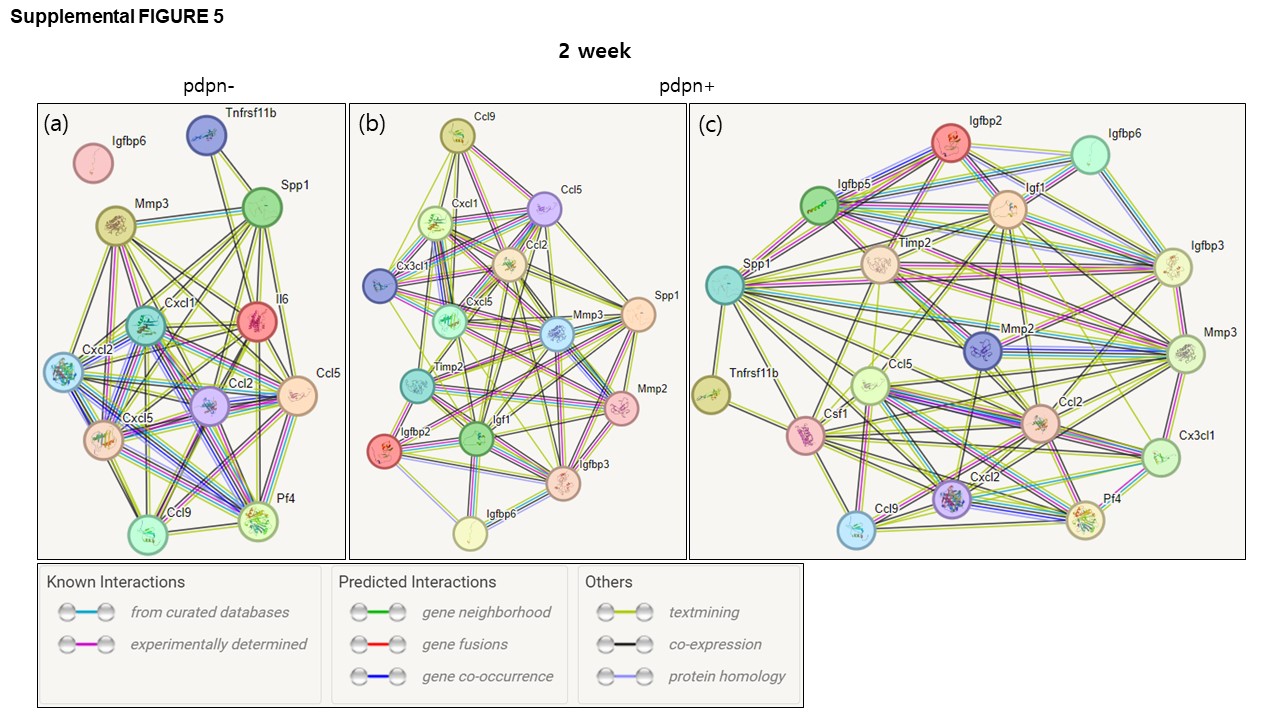


**Supplemental Table 1** Cytokines expression in 1- and 2-week pdpn+/- membranes

| Gene | Pdpn^-^ | |  | Pdpn^+^ | | References | | | |  |
| --- | --- | --- | --- | --- | --- | --- | --- | --- | --- | --- |
|  | 1W | 2W |  | 1W | 2W |  |  |  |  |  |
| CX3CL1  IGFBP3  IGFBP6  IL6  CXCL1/KC  CXCL5/LIX  CCL2/MCP1  CCL9/MIP1γ  RANTES/CCL5  IGFBP2  IGF1  MMP2  MMP3  OPN/SPP1  OPG/TNFRSF11B  TIMP2 | -  -  ++  ++  ++  ++  ++  ++  -  -  -  -  ++  ++  ++  - | -  -  ++  ++  ++  ++  ++  ++  +  -  -  -  ++  ++  ++  - |  | -  -  ++  ++  ++  ++  ++  ++  +  -  ++  ++  ++  ++  ++  - | ++  ++  ++  -  ++  ++  ++  ++  ++  ++  ++  ++  ++  ++  +  ++ | S  S  S  S  S  S  S  S  S  S  S  S  S  S  S | R  R  R  R  R  R  R  R  R  R  R  R  R  R  R | I  I  I  I  I  I  I  I  I  I  I  I  I  I  I  I | O  O  O  O  O  O  O  O  O  O |  |
| 1W=1-week, 2W=2-week, -= low expression, +=mild expression, ++=strong expression  I=inflammation, S=senescence, R=remodeling, O=osteocyte | | | | | | | | | |  |

**Supplemental FIGURES legend**

**Supplemental FIGURE 1** SAβG staining of trabecular and cortical bone next to the growth plates in 8-week C57BL/6 and 12-week SD rats. (a-c) Mouse frozen section samples. (a) SAβG and eosin staining in the femur and tibia. (b) Magnification of the SAβG staining (b) area of (a). (c) Magnification of SAβG stained femur next to the growth plate (c) area of (b) (decline arrows). (d-f) Rat paraffin section samples and SAβG stained cells in the femur and tibia of the trabecular and cortical bone (decline arrows). (d) SAβG and eosin staining in the trabecular bone of the femur. (e) SAβG and eosin staining of the trabecular bone of the tibia. (f) SAβG and eosin staining in the cortical bone of the tibia and femur. Scale bars (a)=1 mm, (b)=0.2 mm, (c)=0.1 mm, (d) and (e)=500㎛, (f)=20㎛. GP=growth plate. TB=trabecular bone. CB=cortical bone.

**Supplemental FIGURE 2** qPCR analysis of the osteocytes and senescence marker genes expression of pdpn^-^ and pdpn^+^ in primary osteocytes cultured for one week before FACS sorting. (a) Podoplanin (Pdpn/E11) expression. (b) DMP1 expression. (c) RANKL expression. (d) SOST expression. (e) p16/CDKN2A expression. (f) p53 expression. P<0.001 ***, NS=no significance.

**Supplemental FIGURE 3** TRAP staining of the osteoclasts in co-cultured osteocytes and BMMs for 3 weeks in 96-well plates. (a) TRAP staining of the osteoclasts co-cultured pdpn- osteocytes and BMMs. (b) TRAP staining of the osteoclasts co-cultured pdpn^+^ osteocytes and BMMs.

**Supplemental FIGURE 4** Multiple protein interactions of the pdpn^+/-^ cytokines after 1-week culture of osteocytes. (a) Very strong expression in pdpn^-^ 1-week membrane. (b) Very strong expression in pdpn^+^ 1-week membrane. (c) Very strong and strong expression in pdpn^+^ 1-week membrane. The protein interactions were analyzed using the STRING database (<https://string-db.org/>). The figure illustrates the complex network of interactions among the cytokines and their potential signaling pathways.

**Supplemental FIGURE 5** Multiple protein interactions of the pdpn^+/-^ cytokines after 2-week culture of osteocytes. (a) Very strong and strong expression in pdpn^-^ 2-week membrane. (b) Very strong expression in pdpn^+^ 2-week membrane. (c) Very strong and strong expression in pdpn^+^ 2-week membrane. The protein interactions were analyzed using the STRING database (<https://string-db.org/>). The figure illustrates the intricate network of interactions among the cytokines, providing insight into their potential roles in cellular communication and signaling.
