# Supplementary material for: Exploring the Interplay Between Senescent Osteocytes and Bone Remodeling in Young Rodents
Source: J Aging Res. 2024 Nov 16;2024:4213141. doi: 10.1155/2024/4213141 (PMC11585373; doi:10.1155/2024/4213141)

Supplemental Figure 1

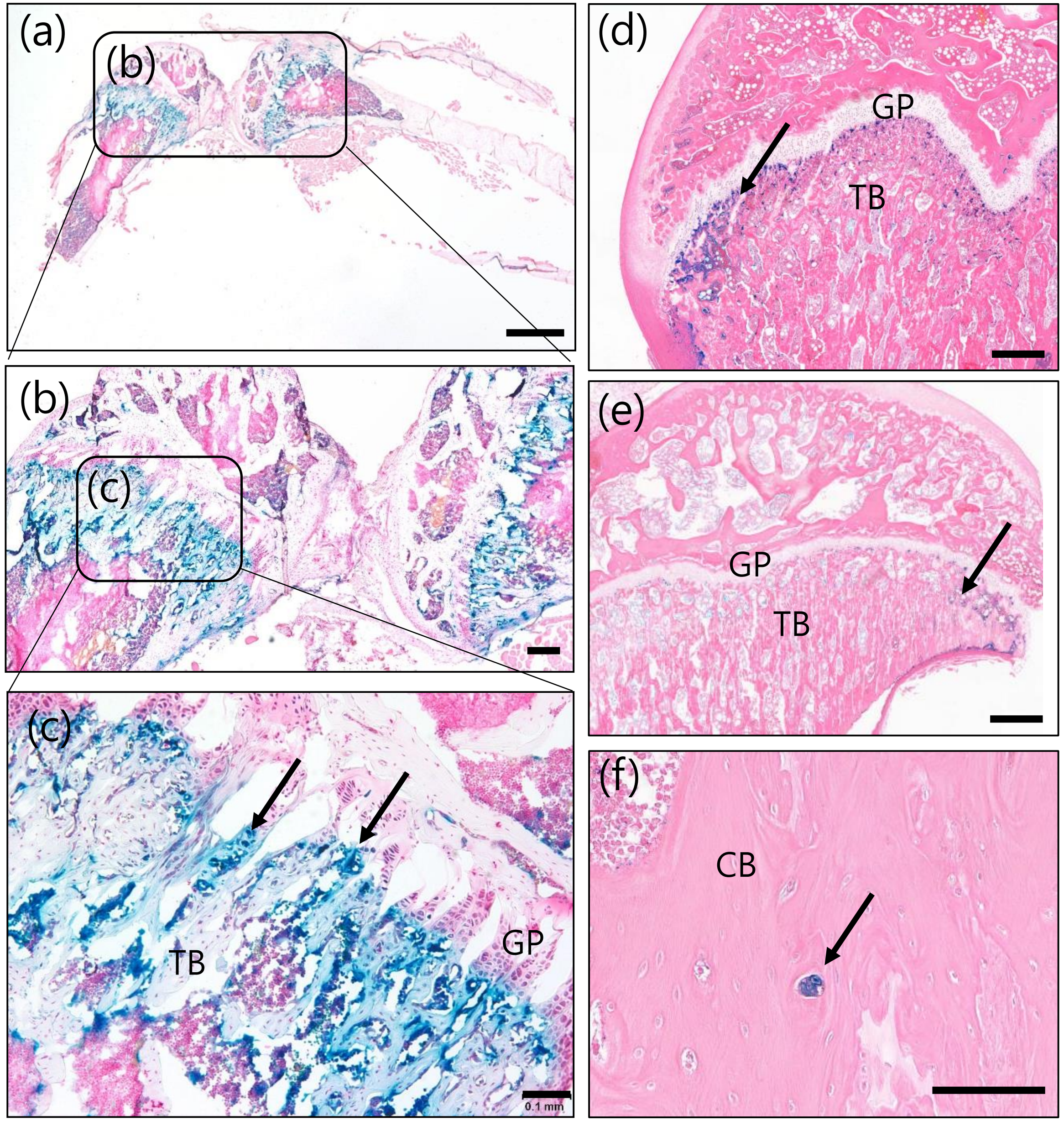

Supplemental FIGURE 2

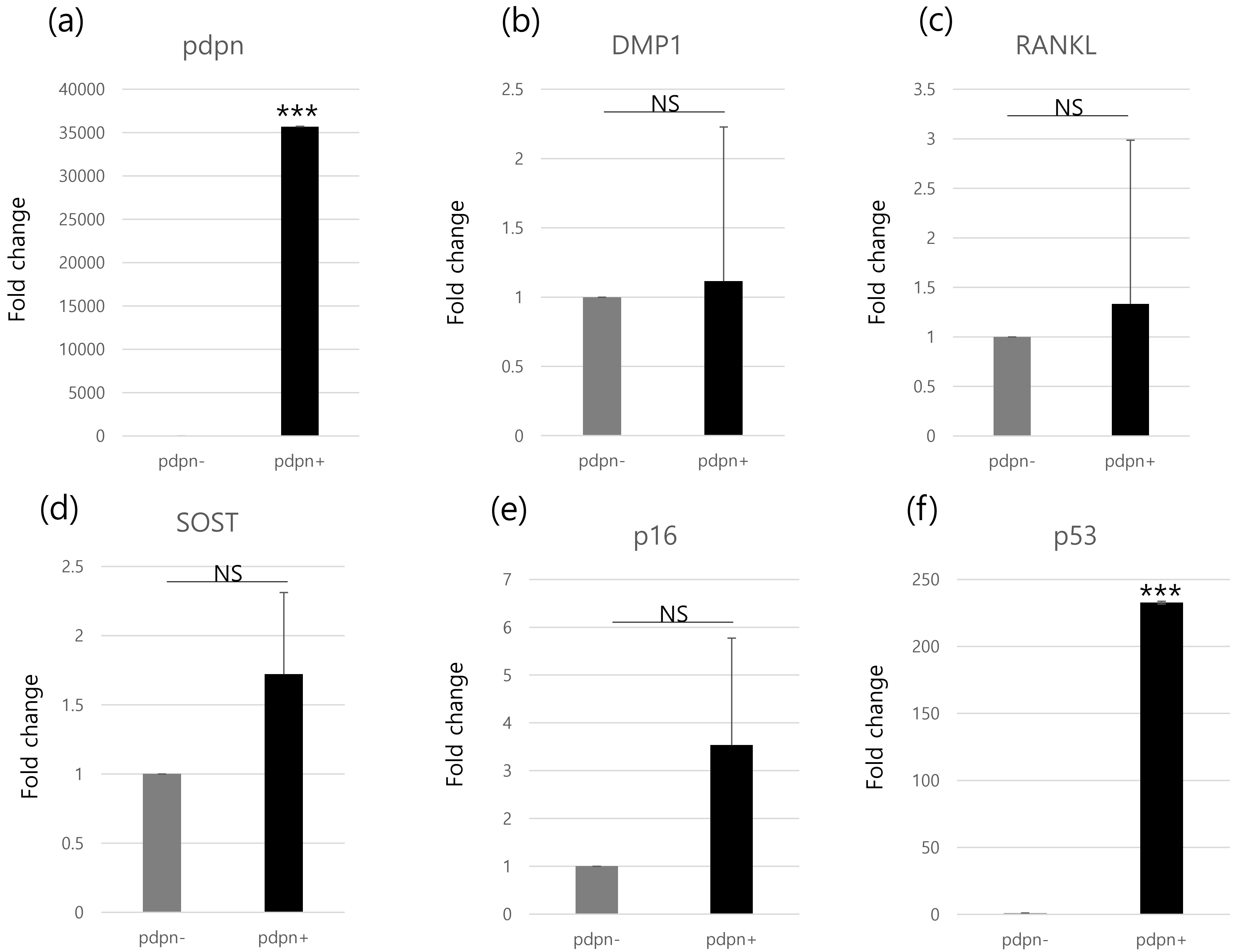

Supplemental Figure 3

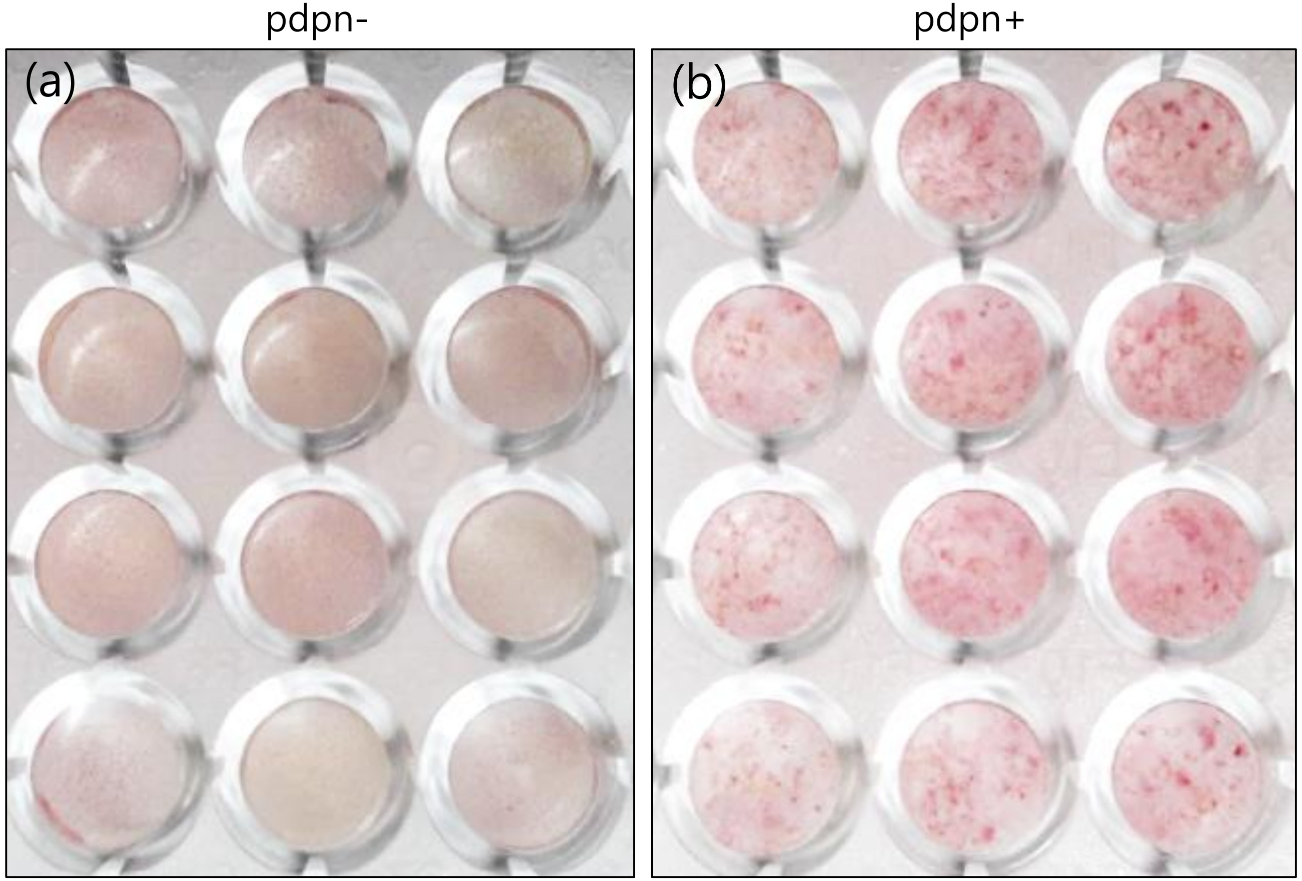

Supplemental Figure 4

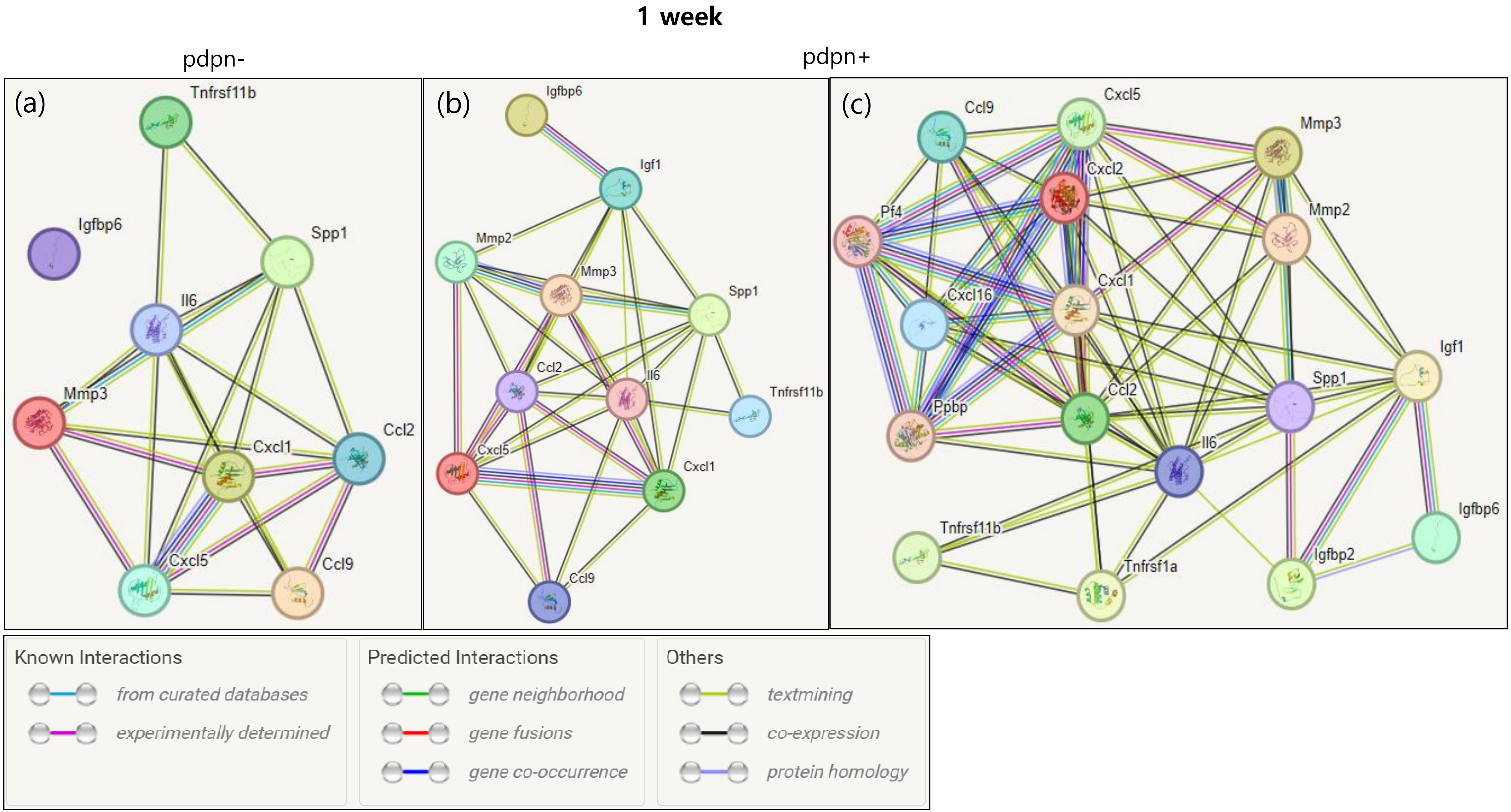

Supplemental FIGURE 4

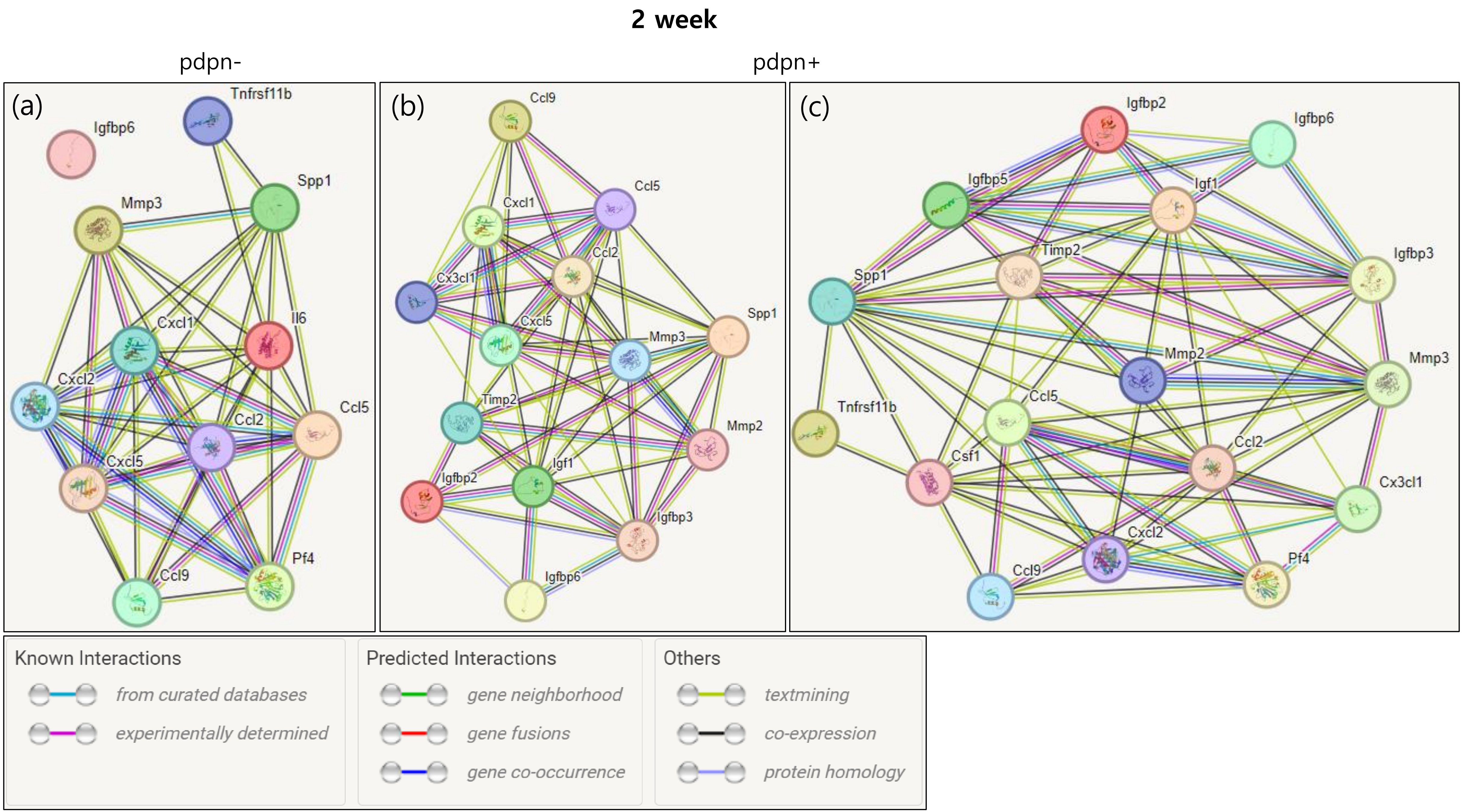

Supplement: Supporting Information — Supporting Figure 1: SAβG staining of trabecular and cortical bone next to the growth plates in 8-week C57BL/6 and 12-week SD rats. Supporting Figure 2: qPCR analysis of the osteocytes and senescence marker genes expression of pdpn− and pdpn+ in primary osteocytes cultured for 1 week before FACS sorting. Supporting Figure 3: TRAP staining of the osteoclasts in co-cultured osteocytes and BMMs for 3 weeks in 96-well plates. Supporting Figure 4: Multiple protein interactions of the pdpn+/− cytokines after 1-week culture of osteocytes. Supporting Figure 5: Multiple protein interactions of the pdpn+/− cytokines after 2-week culture of osteocytes. Table S1: Cytokines expression in 1- and 2-week pdpn ± membranes. [file 4213141.f1.zip › SuppFigures -2024-09-22-JAR.pdf]
